# Antiinflammatory Activity of Melatonin in Central Nervous System

**DOI:** 10.2174/157015910792246155

**Published:** 2010-09

**Authors:** Emanuela Esposito, Salvatore Cuzzocrea

**Affiliations:** 1 Department of Clinical and Experimental Medicine and Pharmacology, School of Medicine, University of Messina, Italy; 2 IRCCS Centro Neurolesi "Bonino-Pulejo", Messina, Italy

**Keywords:** Melatonin, inflammation, neurodegeneration, mitochondria, antioxidant, free radical.

## Abstract

Melatonin is mainly produced in the mammalian pineal gland during the dark phase. Its secretion from the pineal gland has been classically associated with circadian and circanual rhythm regulation. However, melatonin production is not confined exclusively to the pineal gland, but other tissues including retina, Harderian glands, gut, ovary, testes, bone marrow and lens also produce it. Several studies have shown that melatonin reduces chronic and acute inflammation. The immunomodulatory properties of melatonin are well known; it acts on the immune system by regulating cytokine production of immunocompetent cells. Experimental and clinical data showing that melatonin reduces adhesion molecules and pro-inflammatory cytokines and modifies serum inflammatory parameters. As a consequence, melatonin improves the clinical course of illnesses which have an inflammatory etiology. Moreover, experimental evidence supports its actions as a direct and indirect antioxidant, scavenging free radicals, stimulating antioxidant enzymes, enhancing the activities of other antioxidants or protecting other antioxidant enzymes from oxidative damage. Several encouraging clinical studies suggest that melatonin is a neuroprotective molecule in neurodegenerative disorders where brain oxidative damage has been implicated as a common link. In this review, the authors examine the effect of melatonin on several neurological diseases with inflammatory components, including dementia, Alzheimer disease, Parkinson disease, multiple sclerosis, stroke, and brain ischemia/reperfusion but also in traumatic CNS injuries (traumatic brain and spinal cord injury)

## INTRODUCTION

Brain tissue is very vulnerable to free radical damage because of its high oxygen utilization (20% of the total oxygen inspired), high concentrations of polyunsaturated fatty acids [57] and transition metals such as iron, which is involved in the generation of the hydroxyl radical [76], and low concentrations of cytosolic antioxidants [44, 56, 152]. Glutathione (GSH) is the predominant antioxidant in the brain that is present at millimolar concentrations [174]. Melatonin, a secretory product of the pineal gland, is a ubiquitously acting direct free radical scavenger and also an indirect antioxidant [6, 197]. Melatonin, as well as its metabolites, are efficient in detoxifying reactive oxygen species (ROS), e.g., HO• and H2O2 [194], and they also interact directly with reactive nitrogen species (RNS) [32, 68, 146, 196]. Endogenous melatonin is synthesized in the pineal gland from the neurotransmitter serotonin in a circadian manner. In mammals, the melatonin rhythm is generated by an endogenous circadian master clock in the suprachiasmatic nucleus (SCN) of the hypothalamus, which is entrained by the light/dark cycle over a 24-hr period. It participates in several neuroendocrine and physiological processes and is appropriately referred to as a tissue factor, a paracoid, an autocoid, and an anti-oxidant and sometimes as a hormone depending on its physiological actions [192]. It has profoundanti-oxidant actions against oxidative and nitrosative stress [159]. It also exerts significant immunomodulatory influences [189] and modulates angiogenesis and wound healing processes [181]. Melatonin exerts its effects through receptor-mediated and receptor-independent mechanisms, thus, manifesting enormous functional versatility and diversity [160]. The ubiquitous distribution of melatonin receptors in the CNS as well as in the peripheral organs further compliments the fact that melatonin’s actions are not compromised by morpho-physiological barriers such as the blood-brain barrier. It is likely to influence every cell with which it comes into contact. At physiological concentrations, melatonin exerts receptor-mediated actions whereas the receptor-independent actions usually require higher supraphysiological/pharmacological melatonin concentrations because of the circumstances under which it is tested. Even at the higher concentrations, melatonin is well-tolerated by humans [176]. Two melatonin receptors, MT1 and MT2, which belong to the G-protein-coupled receptor superfamily, have been cloned in mammals and share some specific short amino-acid sequences, suggesting that they represent a specific subfamily. A third melatonin-binding site has been purified and characterized as the enzyme quinine reductase 2, and the inhibition of this enzyme by melatonin may contribute to its anti-oxidant properties [138, 195]. 

Exogenous administration of melatonin can also entrain the circardian clock by a direct action on SCN and, thus, it has potential to treat the disoriented circardian clock in cases such as jet-lag, shift-work, in profoundly blind subjects, and in individuals with delayed or advanced sleep phase syndromes and sleep inefficiency [72]. Besides being used to increase sleep efficiency, treat jet lag, improve the cardiovascular system [177], and as an antiaging drug [21, 71, 167] and a dietary supplement and cancerprotective hormone [149], intensive research has indicated melatonin’s beneficial effects in experimental models of neurodegenerative disorders. Brain oxidative damage has been implicated as a common link in the pathogenesis of such diseases. 

## MELATONIN AND ITS RECEPTORS

Melatonin (*N*-acetyl-5-methoxytryptamine) is a natural hormone secreted by the pineal gland. In clinical use for many years, melatonin is safe and well-tolerated even at high doses [214]. Melatonin, or N-acetyl-5-methoxytryptamine, is an indole mainly produced in the mammalian pineal gland during the dark phase. Melatonin secretion from the pineal gland has been classically associated with circadian and circanual rhythm regulation, and with adjustments of physiology of animals to seasonal environmental changes [151]. Melatonin production, however, is not confined exclusively to the pineal gland, but other organs and tissues including retina, Harderian glands, gut, ovary, testes, bone marrow and lens also have been reported to produce it [128, 193]. In recent years, it has become apparent that melatonin’s actions transcended those of a hormonal modulator. Melatonin had been shown to subtly influence the function of a variety of tissues and cells not generally considered in the endocrine category [193]. This led to a suspicion that melatonin had yet undefined actions at the cellular level. Numerous data suggest a role for melatonin and its metabolites function in the antioxidative defence in all organisms that produce this indole [120, 158, 168, 196], particularly, since melatonin crosses all morphophysiological barriers and enters all cells. Several mechanisms have been proposed for the neuroprotective effect of melatonin. Melatonin upregulates antioxidative defensive systems, including the activities of superoxide dismutase and glutathione peroxidase as well as the levels of glutathione [202]. Furthermore, melatonin and its metabolites scavenge free radicals [147, 190] and thus terminate the initiation and propagation of lipid peroxidation. A recent studies show that melatonin binds several metals, including iron [107, 108] and this effect may attenuate the Fenton reaction and the generation of the hydroxyl radical. 

It participates crucially in neurogenesis, immunomodulation [182, 200], improving immune defense [118], regulating circadian rhythms and sleep [1], eliminating free radicals [146, 155, 156, 196], intervening in lipid metabolism [119, 173], and also inhibiting cancer growth [61]. Mechanistically, melatonin functions, by acting through G-protein coupled membrane receptors, MT1 and MT2 [61, 119, 173] and through nuclear receptors RZR/ROR [142]. 

Melatonin also stimulates a host of antioxidative enzymes including SOD, glutathione peroxidase (GPx) and glutathione reductase (GRd); these actions further reduce the oxidation state of cells [6, 122].

Melatonin inhibits the activity of nitric oxide synthase and dopamine release, potentiates the inhibitory effect of GABA in the central nervous system, modulates the serotonin receptors and potentiates the opioid analgesic. 

Melatonin possesses an electron-rich aromatic indole ring and functions as an electron-donor, thereby reducing and repairing electrophilic radicals [124]. Of additional interest regarding melatonin, is its reported binding to quinone reductase 2 [217]. This enzyme, which is considered a melatonin receptor, is important in the detoxification of pro-oxidant quinones. Experimental evidence supports its actions as an indirect antioxidant when stimulating antioxidant enzymes [161], its ability to enhance the activities of other antioxidants and its protection of antioxidant enzymes from oxidative damage [126].

Melatonin has two important functional groups which determine its specificity and amphiphilicity: the 5-methoxy group and the N-acetyl side chain. Once synthesized, the majority of melatonin diffuses directly towards the cerebrospinal fluid of the brain's third ventricle, while another fraction is released into the blood stream where it is distributed to all tissues [33]. Circulating melatonin is metabolized by P-450 liver enzymes, which hydroxylate this hormone at the 6-carbon position to yield 6- hydroxymelatonin. This reaction is followed by conjugation with sulfuric or glucuronic acid, to produce the principal urinary metabolite, 6-sulfatoxymelatonin. In the last step, conjugated melatonin and minute quantities of unmetabolized melatonin are eliminated through the kidney. In addition to hepatic metabolism, oxidative pyrrole-ring cleavage appears to be the major metabolic pathway in other tissues, including the CNS [163]. Melatonin exhibits high affinity binding to its receptors, of less than 500 pM [77]. There are four different melatonin receptor subtypes. Two of them are membrane-associated receptors, while the other two are nuclear receptors. Membrane melatonin receptors are classified based upon their kinetic properties and pharmacological profiles into MT1 and MT2 melatonin receptor subtypes [186]. They belong to the seven transmembrane receptor family, have 60% aminoacid homology, and differ in molecular structure and gene chromosomal localization [162]. MT1 andMT2 melatonin receptor subtypes are present in humans and other mammals [162]. The MT2 melatonin receptor has lower affinity (Kd=160 pmol/l) for ^125^I-melatonin as compared to the human MT1 melatonin receptor (Kd=20-40 pmol/l) [186]. Although several authors proposed the existence of a putative MT3 melatonin receptor subtype, recent studies have provided evidence that the MT3 melatonin receptor is actually a cytosolic quinine reductase 2 enzyme rather than a membrane receptor [117]. For this reason, the MT3 melatonin receptor subtype is no longer recognized in IUPHAR's classification as a G protein-coupled melatonin receptor subtype. Melatonin is also a ligand for retinoid orphan nuclear hormone receptors [12], referred to as RZRα and RZRβ at concentrations in the low nanomolar range. Both receptors are present in the central and peripheral nervous system and have been associated with cell differentiation and immune response regulation [179]. The RZRα melatonin receptor has been implicated in inflammatory reactions. Thus, Steinhilber *et al*. [183] reported that melatonin can down-regulate the expression of 5-lipooxygenase (5-LOX), an important inflammatory mediator, in B lymphocytes *via *RZRα receptors. 

Several groups have shown that melatonin reverses chronic and acute inflammation [37, 40]. Melatonin treatment also causes an important reduction of nitric oxide (NO) and malondialdehyde (MDA) levels, two compounds that are closely related to inflammation [15]. Communication between the brain and the immune system involves a complex network of bidirectional signals which link the neural, endocrine, and immune systems [104, 131]. Cerebral disorders disrupt the functional interaction between the nervous and immune systems [27]. The immunomodulatory properties of melatonin are well known; it acts on the immune system by regulating cytokine production of immunocompetent cells [26, 67]. Experimental and clinical data showing that melatonin reduces adhesion molecules and pro-inflammatory cytokines including IL-6, IL-8, and tumor necrosis factor (TNF)-α and modifies serum inflammatory parameters. As a consequence, melatonin improves the clinical course of illnesses which have an inflammatory etiology [148]. 

**Fig. (1):**
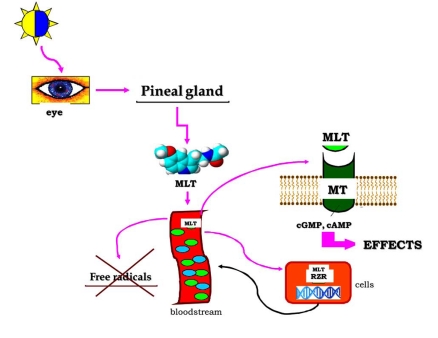
The regulative pathway of melatonin synthesis in pineal gland and the action sites of melatonin in biological system.

Melatonin has several additional anti-inflammatory effects, which are probably related to a direct interaction with specific binding sites located in lymphocytes and macrophages [141] (Fig. **1**). 

## EFFECT OF MELATONIN ON NEURODEGENERATIVE DISORDERS

The inherent biochemical and physiological characteristics of the brain, including high polyunsaturated fatty acids and energy requirements, make it particularly susceptible to free radicals mediated insult [23]. Increasing evidence indicates that accumulation of aberrant or misfolded proteins, protofibril formation, ubiquitin proteasome system dysfunction, excitotoxic insult, oxidative and nitrosative stress, mitochondrial injury and failure of axonal and dendritic transport represent unifying events in many slowly progressive neurodegenerative disorders [171]. 

Several effects of melatonin through its receptors may account for its ability to prevent oligodendroglial damage: free radical scavenger production by activated microglia [130, 196], improvement of membrane fluidity and reduction of edema and polymorphonuclear cell infiltration into damaged tissue, prevention of translocation of the nuclear factor-kappa-B (NF-κB) to the nucleus and the subsequent reduction of pro-inflammatory cytokines expression, which play a relevant role in the inflammatory reaction [123, 125]. In addition, melatonin could modulate astrocyte reactivity or death through an upregulation of astrocytic anti-oxidative defenses [22]. The neuroprotective pathway appears to implicate both melatonin receptors and inflammatory modulation, leading to a promotion of oligodendrocyte maturation.

NO plays crucial roles in the brain such as neuromodulation, neurotransmission and synaptic plasticity, but is also involved in pathological processes including neurodegeneration and neuroinflammation [62]. It is now well documented that NO and its toxic metabolites can inhibit components of the mitochondrial respiratory chain leading to cellular energy deficiency and, eventually, to cell death [46]. Astroglial cells become “activated” in a wide range of CNS pathologies, leading to the induction of iNOS [17, 48]. Although glial activation can be protective, excess activation causes damage in most occasions [133] since the NO formed may inhibit neuronal respiration. Formation of NO by astrocytes has been suggested to contribute, *via *impairment of mitochondrial function, to the neurodegenerative process. In addition, a relationship between neuronal injury and NO originated from glial cells has been suggested [41] since activation of iNOS results in the release of large amounts of NO neurotoxic over long periods of time [121, 222]. Several possibilities have been proposed for neurodegenerative damage in CNS, including oxidative stress. Astrocytes are involved in multiple brain functions in physiological conditions participating in neuronal development, synaptic activity and they also actively participate in the processes triggered by brain injuries, aimed at limiting and repairing brain damage [47]. Reactive astrocytes have been implicated in several neurological diseases with inflammatory components, including HIV-1- associated dementia, Alzheimer disease (AD), multiple sclerosis and stroke [69, 129]. Astrocytes contribute to the neuroprotection and survival of neurons; any astrocytic dysfunction seriously affects neuronal viability. In addition, astrocytes preserve neuronal survival through inactivation of ROS. Glia become activated by inflammatory mediators and express new proteins such as the iNOS [121]. NO produced by iNOS seems to be a key mediator of such glial-induced neuronal death.

In recent decades, neuroinflammation has been proposed as a causative factor of neurological diseases and disorders [200]. The chronic inflammatory state would create an activated immune response, including acute phase protein response, cytokines (interferons and interleukins), macrophages, lymphocytes, and other immune system cells as well [191]. Oxidative stress in the brain occurs when the generation of ROS exceeds the ability of the endogenous antioxidant system to remove excess ROS subsequently leading to cellular damage. The presence of oxidative stress in brain mitochondria should cause changes in LPO and GSH and, if ROS damage is prolonged, also changes should occur in enzymes of the redox cycle of GSH [122].

Melatonin, with antioxidant and anti-inflammatory well documented actions [32, 83, 146, 165, 166, 190, 197], showed marked beneficial effects against brain mitochondrial dysfunction with age. These effects, and the fact that melatonin virtually lacks toxicity even at large doses [42, 84, 114], supports its clinical use in preventing the impairments of aging. Due to the lipophilicity of melatonin, it is accumulated by the mitochondria at high concentrations [36], allowing its interaction with mitochondrial membranes [150], possibly locating near the polar heads of membrane phospholipids [90]. In this location, melatonin may easily protect brain mitochondrial membranes from free radical attack, stabilizing them [102]. The ability of melatonin to prevent GSH loss with age probably reflects its effect on the activities of the GSH redox cycle enzymes [122]. 

Mitochondria have been identified as a target for melatonin [5, 92]. Melatonin promotes mitochondrial homeostasis. Melatonin may be possible to treat neurodegenerative disorders by inhibiting mitochondrial cell death pathways [82, 112, 180, 205]. Studies show that melatonin levels are lower in AD patients compared with that in age-matched control subjects [112, 116, 140]. The great advance has been currently conduced in studies of protection against AD by antioxidant melatonin inhibiting A*β*-induced toxicity [52-55, 85, 100, 225] and attenuating tau hyperphosphorylation [43, 105, 113, 143, 210, 211, 213]. Besides the antioxidant properties, the anti-amyloidogenic properties of melatonin for AD have been studied [86, 144]. Melatonin improved learning and memory deficits in an APP695 transgenic mouse model of AD *in vivo *[52]. *In vitro *experiments showed that A*β*-treated cultures exhibited characteristic features of apoptosis, and melatonin attenuated A*β*-induced apoptosis in a number of cellular models of AD including mouse microglial BV2 cells, rat astroglioma C6 cells, and PC12 cells [43, 52, 54, 145, 225]. Studies in transgenic AD mice and cultured cells have suggested that administration of melatonin inhibited the A*β*-induced increase in the levels of mitochondria related Bax [43, 55]. Furthermore, melatonin prevented upregulated expression of Par-4 and suppressed A*β*-induced caspase-3 activity [55]. Another experiment in mouse microglial BV2 cells *in vitro *showed that melatonin also decreased caspase-3 activity, inhibited NF-*κ*B activation, and reduced the generation of A*β*-induced intracellular ROS (reactive oxygen species) [53]. In addition, *in vivo *observations showed that melatonin-treated animals had diminished expression of NF-*κ*B compared to untreated animals [209]. Another experiment demonstrated that melatonin inhibited the phosphorylation of nicotinamide adenine dinucleotide phosphate (NADPH) oxidase *via *a PI3K/Akt-dependent signaling pathway in microglia exposed to A*β*1-42 [100]. Taken together, the above-mentioned evidence suggests that melatonin may provide an effective means of treatment for AD through its antiapoptotic activities.

Mounting evidence indicates that melatonin blocks the 1-Methyl-4-phenyl-1, 2, 3, 6-tetrahydropyridine (MPTP)-dependent apoptotic fragmentation of nuclear DNA in rat astrocytes [89], rat mesencephalic cultures [80], and mouse striatal neurons [81]. Besides its traditional role as an antioxidant and free radical scavenger, melatonin proved to target a variety of pathways while its systemic effect correlates with the drug’s disruption of the intrinsic mitochondrial cell death pathway, silencing of the Rip2/caspase-1 pathway, and the activation of survival pathways. These actions may be synchronistic and complementary in models of HD. Effective treatment to prevent neurodegeneration could be achieved using a combination of melatonin and other pharmacological agents that act on different apoptosis targets.

Antiinflammatory actions of melatonin depend on its inhibition of the expression of iNOS and, here, mitochondrial iNOS [38, 49]. It was recently reported that the brain melatonin metabolite N1-acetyl-5-methoxykynuramine (AMK) [163] is a better antioxidant than its precursor AFMK [164], and it is a highly efficient NO scavenger, which forms a stable product that does not easily re-donate NO [73]. AMK was more potent than melatonin in inhibiting *in vitro* and *in vivo* striatal NOS activity [103], and both compounds, melatonin and AMK, easily cross the brain-blood barrier after their administration, reaching neuronal and glial cells [16, 19, 103]. Moreover, several of the melatonin metabolites, in addition to AMK, and including 3-hydroxymelatonin, and AFMK are likewise free radical scavengers [146, 196]. With regard to the anti-inflammatory effects of melatonin, the most important feature is its inhibition of iNOS expression [38]. Antioxidant and anti-inflammatory properties of melatonin are relevant in mitochondrial physiology [16], and they may play a neuroprotective role in PD. AMK is a potent inhibitor of mitochondrial iNOS activity and a more efficient NO scavenger than its precursor melatonin [73]. 

Melatonin is known to be a stabilizer or protector of cell and organelle membranes because of its inhibitory effects on lipid peroxidation [153]. Melatonin has a regulatory effect on the activities of enzymes involved in the generation of free radicals, and on microsomal membrane fluidity in situations of elevated oxidative stress [208]. Moreover, its solubility in lipid and aqueous media, which allows it to cross morphophysiological barriers and enter subcellular compartments, permit melatonin to function as a highly effective inhibitor of oxidative damage. Our results indicate that melatonin stabilizes C6 glioma cell line membranes. Thus, melatonin’s ability to stabilize membranes may contribute to its neuroprotective actions. Recent work shows that melatonin prevents specifically the activation of the pro-inflammatory enzymes COX-2 and iNOS in glioma cells without simultaneous inhibition of COX-1 enzyme, thus indicating an anti-inflammatory action. In fact, COX-2 inhibition by melatonin may account for many protective effects including those observed in neurodegenerative diseases. Importantly, melatonin did not alter COX-1 protein level, one of the major disadvantages of using NSAIDs is their side effect on COX-1 [66]. Due to its lack of effect on COX-1, melatonin could potentially share the benefits of NSAIDs while avoiding their side effects. Moreover, melatonin lowered the nitrite/nitrate production by reducing iNOS expression in LPS/IFN-γ through the inhibition of NF-κB activation. Inhibition of NF-κB by melatonin has been previously reported in other experimental models that show how melatonin prevents NF-κB activation by oxidative stress [24]. Moreover, our study showed an inhibitory effect of melatonin on nNOS expression in glioma cell line, confirming a role of noncompetitive inhibitors of the nNOS. Additional studies are required to define the specific concentrations of melatonin to inhibit oxidative damage in specific disease/conditions. Melatonin protects against NO-induced impairment and this effect is associated with decreased formation of oxidatively modified proteins and with decreased upregulation oxidative stress-responsive genes, such as Hsp70. Expression of stress responsive genes, such Hsp70, used as indicator of astroglial stress, is markedly inhibited, consistent with a protective effect of melatonin. 

## EFFECT OF MELATONIN ON TRAUMATIC CNS INJURIES

Traumatic CNS injuries include traumatic brain injury (TBI) and SCI, depending on the anatomical region damaged. It is crucial to relate melatonin’s efficacy to the aberrant Ca2+-homeostasis-driven signaling pathways as they form a common denominator to any traumatic CNS injury. Melatonin is highly effective in preventing molecular mutilation due to aberrant Ca2+-homeostasis. A recent experimental study in aged mice found that oral administration of melatonin restored the metabolic function of cells with improvement in several aspects of Ca2+-signaling such as the amplitude and frequency, the size of intracellular Ca2+-pools, capacitative Ca2+-entry, and the mitochondrial potential [102, 202]. Such aberrant Ca2+-homeostasis during aging is undoubtedly a slow and gradual process that causes the accumulation of molecular debris over time. Some benefits of melatonin may also be expected when sudden neurotrauma disturbs tightly regulated Ca2+-signaling processes. This evidence comes from the experimental studies where melatonin alleviated the impaired large conductance of Ca2+-activated K+ channel activity in hippocampal neurons, which were injured as a result of intermittent hypoxia [201] or in ischemia-reperfusion injury in chronically hypoxic rats [219]. Melatonin receptors, in addition to coupling with the heterotrimeric G proteins, also physically associate with other intracellular proteins, e.g., calmodulin [141], and such interactions multiply the modulatory functions of melatonin in cell signaling [87] and cytoskeltal rearrangements [13, 79]. Moreover, melatonin receptors undergo heterodimerization as MT1/MT2, although the functional consequences of this association on receptor signaling and trafficking are currently unknown [87]. The advantages of therapeutic and clinical utilization of melatonin have been repeatedly emphasized [2, 99] and such encouragement may, in due course, lead to an efficient neuroprotective intervention in CNS trauma. One may expect dual benefits of melatonin administration in traumatic CNS injuries. Firstly, it may be important to restore the perturbed endogenous melatonin rhythm if it is disturbed by mechanical interruption of melatonin synthesis due to damage to the neural connections between the SCN and the pineal gland. Secondly, it may be a result of its multiple neuroprotective/neurorestorative actions that melatonin has at the site of injury in the CNS. Progression of traumatic CNS injuries follow an archetypal course through primary and secondary damaging events, which are distinct in their spatiotemporal windows. Because of melatonin’s multiple actions, its use as a treatment may profoundly influence both the short-term primary damaging events as well as preventing some of the long-term secondary damage. The half-life of exogenously administered melatonin in the blood is short; hence, there is a need for stable melatonin mimetics, be they synthetic ligands or receptor modulators. To date, these melatonin analogs have not been tested for their possible benefits in SCI or any CNS injury.

Clinically, SCI involves two components: an initial mechanical instability precipitating into a secondary injury process leading to the final neurological deficit [109]. Traumatic SCI may be a direct lesion that causes focal injury to the neural elements at the site of impact or a lesion due to stretching or compressive forces applied to spinal cord *via *bones and/or ligaments. In either case, the cord lesion expands with time following injury over adjoining spinal segments causing secondary injury. Primary injuries cause disruption of the blood supply to spinal cord and lead to ischemic damage. A quest for a good neuroprotectant in SCI remains unresolved. A possible correlation between the neuroprotective efficacy of melatonin and SCI originally emerged almost three decades ago as clinical case reports in which the circardian profiles of endogenous melatonin were assessed in SCI patients. Much later the experimental studies on the utility of exogenously-applied melatonin in experimental models of SCI were conducted. 

Levels of melatonin in SCI patients differed strictly with respect to the site of injury. SCI disrupting the cervical spinal cord significantly perturbed the levels of endogenous melatonin, whereas, an injury at the levels of thoracic or lumbar did not affect it severely. These differences are explicable in terms of the central neural pathways, which connect the eyes to the pineal gland. Lesions within the cervical spinal column caused decentralization of pineal gland because they perturbed descending sympathetic fibers and led to an absence of significant increment in nocturnal melatonin; this, clearly distinguished quadriplegic subjects from normal males and from the subject with a lesion of the lumbar spinal cord [96]. It was further confirmed that neurologically complete cervical spinal cord transection results in complete loss of the circardian melatonin rhythm [223]. More complete studies on rhythms of serum melatonin in patients with spinal lesions at the cervical, thoracic, or lumbar region revealed that the cervical region of the spinal cord includes the neural pathway, which is essential for the diurnal rhythm of pineal melatonin secretion in human. Retrospectively, such studies clearly discerned the relationship between regional specificity of SCI lesions and changes in the endogenous profile of melatonin [106]. Such a regional bias was reconfirmed in a recent clinical study conducted using tetraplegic patients with bilateral oculo-sympathetic paresis showing a complete loss of the nocturnal production of melatonin [224]. A recent assessment based on the “Basic Nordic Sleep Questionaire" conducted at the Karolinska University Hospital in Stockholm, on a large SCI patient population comprised of 230 patients, inferred that perturbances in the melatonin rhythm contributed to poor subjective sleep quality associated with higher ratings of pain intensity, anxiety, and depression [137]. Another study confirmed the positive correlation of an altered melatonin cycle in cervical SCI patients with reduced sleep quality and documented the need for larger studies on the potential usefulness of melatonin replacement therapy in normalizing sleep in SCI patients [175]. Several encouraging clinical case studies suggest that melatonin may be the neuroprotectant of choice in this devastating injury. There is no neuroprotective drug in clinical practice to date that is highly useful as a treatment for this complex neurological problem. Hence, it is important to assess the outcomes of melatonin’s use in experimental models of SCI with the intent of eventually applying this information for treatment of SCI in humans. The first experimental study to test melatonin’s efficacy in reducing neural damage in experimental SCI in animals is that of Fujimoto and colleagues [59]. They studied an acute to chronic SCI model and showed a significant protection by melatonin against neutrophil-mediated damage including lipid peroxidation, reduced levels of secondary injury, and an earlier recovery [59]. Systemically applied pharmacological doses of melatonin were shown to boost the antioxidant defense system in a variety of ways after acute SCI [198]. Melatonin also protected against autodestruction following SCI by reducing the levels of free iron and the products of lipid peroxidation [111]. When compared with methylprednisolone, melatonin was found to be more effective in an acute SCI model against lipid peroxidation and in preserving the structural integrity of neurons, axons, and intracellular organelles [91]. A later study uncovered the greater efficacy of melatonin compared to methylprednisolone in preserving the ultrastructural histopathological integrity of the spinal cord although the reduction of lipid peroxidation after SCI was less obvious. Furthermore, this study also verified that the neuroprotective effect of melatonin was dose-dependent [70]. A combination therapy of melatonin with methylprednisolone did show an additive effect against the accumulation of lipid peroxidation products in the subacute phase of injury, but did not show any difference in a brief chronic 10-day study following SCI in rats as evidenced by neurobehavioral, ultrastructural, and electrophysiological recovery [29]. However, more recently in a SCI model in mice, the combination therapy with melatonin and dexamethasone had a significant and important beneficial anti-inflammatory effect by blocking the possible progression of secondary injury events after SCI. This study showed that the effective dose of dexamethasone could be reduced 10-fold when it was given in combination with melatonin [63]. In comparison to other anti-oxidants such as oxytetracycline or prostaglandin E1, melatonin was found to be more potent in ameliorating secondary damage in an acute model of SCI [203]. Melatonin reduces the development of inflammation and tissue injury associated with SCI by blocking both oxidative and nitrosative stress [65]. Moreover, melatonin limits the expression and activity of matrix metalloproteinases (MMP-9 and MMP-2) thereby also reducing pro-inflammatory TNF-α expression in a mouse model of traumatic SCI [51]. Moreover, melatonin’s protective role in SCI was related to the regulation of MAPK signaling pathways and the high-mobility group box 1 protein expression (HMGB1) in mice. Melatonin treatment in SCI mice enhanced motor recovery, reduced the activation of p38 MAPK, JNK, and ERK1/2, and the expression of HMGB1 [50]. An earlier report also showed that activation of the endogenous melatonin system in the spinal cord reduced the generation, development, and maintenance of central sensitization, with a resultant inhibition of capsaicin-induced secondary mechanical allodynia and hyperalgesia [204]. It was recently confirmed in an experimental model of SCI that exogenously administered melatonin reduced mechanical allodynia by altering the expression of water channel aquaporins [134]. Complementing the data regarding the neuroprotective effects in rodent SCI models, the beneficial neurobiological effects of melatonin have also been demonstrated in the rabbit SCI models. Further confirmation of neuroprotective efficacy of melatonin in traumatic SCI comes from a study conducted in rabbits wherein pinealectomy retarded the recovery rate after SCI, and administration of melatonin exogenously to the pinealectomized animals augmented the recovery [7]. The multifunctional efficacy of melatonin treatment after SCI has been shown *in vivo* by many investigations [172]. Calpain, a Ca2+-dependent neutral protease, is known to be a key player in the pathogenesis of SCI. Treatment of SCI animals with melatonin attenuated calpain expression, inflammation, axonal damage, and neuronal death, indicating that melatonin was highly neuroprotective in this situation. Moreover, examination of levels of calpain and caspase-3 expression and activity indicated significant reductions in the proteolytic events in SCI animals after treatment with melatonin. 

Neutrophils are the first leukocytes to arrive within the injured spinal cord [74, 127]. In this setting, neutrophils release neutral proteases and ROS [39]. In this regard, the production of ROS such as superoxide anions, hydrogen peroxide, and peroxynitrite, is also associated with local and systemic inflammatory response as well as the neurodegenerative disease [157]. Glucocorticoids (GC) are widely used in the management of inflammatory diseases. Glucocorticoids exert beneficial effects after acute CNS injury in humans and experimental animals [94]. Important data show that GC may have a disease-modifying effect in addition to their well-documented anti-inflammatory actions [18]. In fact, the therapeutic management of long-term pathologies with steroids is often linked to a series of unwanted side effects, involving the hypothalamus-pituitary-adrenal axis, the cardiovascular system, as well as the fat and bone metabolism [8]. Glucocorticoids are among a variety of endogenous compounds that have been suggested to influence melatonin production in various vertebrate species, including humans, and the existence of the mutual relationship between the pineal gland and the hypothalamo-pituitary-adrenal axis has been postulated. This study provides the first evidence that the combination therapy with melatonin (10 mg/kg) and dexamethasone (0.025 mg/kg), used at a dose which are not effective when administered as single treatment, attenuates: (i) the tissue damage, (ii) the infiltration of the spinal cord with PMN, (iii) TNF-a expression, (iv) the nitration of tyrosine residues, (v) iNOS expression, (vi) apoptosis level, and (vii) motor recovery. The anti-inflammatory action observed with the combination therapy is also related to the reduction of the degree of iNOS protein. In addition, the degree of staining for nitrotyrosine was significantly reduced by the treatment with the combination therapy in SCI-treated mice. In this study, it was shown that the pharmacologic combination of melatonin plus dexamethasone reduced the inflammatory cell infiltration as assessed by the specific granulocyte enzyme MPO. Neutrophils, recruited into the tissue [199], can contribute to tissue destruction by the production of reactive oxygen metabolites [31, 127], granule enzymes, and cytokines that further amplify the inflammatory response [206]. Already, within 1 hr after SCI, an increased synthesis and/or secretion of TNF-α, is detectable at the injury site [11, 95, 222]. After SCI, TNF-α might serve as an external signal, initiating apoptosis in neurons and oligodendrocytes [10, 34]. It is well known that Bax, a proapoptotic gene, plays an important role in developmental cell death [9] and in CNS injury [135]. Similarly, it was shown that the administration of Bcl-xL fusion protein (Bcl-xL FP; Bcl-2 is the most expressed antiapoptotic molecule in adult central nervous system) into injured spinal cords significantly increased neuronal survival, suggesting that SCI-induced changes in Bcl-xL contributed considerably to neuronal death [115]. The co-treatment of melatonin with dexamethasone in SCI experimental model record features of apoptotic cell death after SCI, suggesting that protection from apoptosis may be a prerequisite for regenerative approaches to SCI. In particular, the co-administration of melatonin with dexamethasone reduced Bax expression, while Bcl-2 protein was expressed much more in SCI treated mice. This result suggests that melatonin with dexamethasone prevents the loss of the antiapoptotic way and reduces the pro-apoptotic pathway activation with a mechanism still to be discovered. Fas (CD95)-mediated apoptosis is an essential mechanism for the maintenance of homeostasis, and the disruption of this death pathway contributes to many human diseases [28]. Results from our research group, moreover, suggest that co-treatment in the acute phase of SCI may decrease the extent of intramedullary spinal cord hemorrhage and damage likely is demonstrated by the histologic tissue analysis. Thus, we have found in the post-traumatic inflammatory combination therapy that melatonin plus dexamethasone exerts a significant and important beneficial anti-inflammatory effect by blocking the possible progression of secondary damage. Furthermore, our results suggest that *in vivo*, the combination therapy allowed reducting 10-fold the effective dexamethasone dose. These findings indicate a novel function of a possible combination therapy, which provides a key procedure for the control of the secondary damage development after SCI. 

SCI is a highly debilitating pathology. Although innovative medical care has improved patient outcome, advances in pharmacotherapy for the purpose of limiting neuronal injury and promoting regeneration are limited. The complex pathophysiology of SCI may explain the difficulty in finding a suitable therapy [185]. It is known that SCI initiates a series of cellular and molecular cascade events [10, 75, 199] and that a progressive neuronal injury results from a combination of secondary injury factors including ischemia, biochemical alterations, apoptosis, excitotoxicity, neurotransmitter accumulation and lipid peroxidation/free radical injury [25]. It is believed that inflammatory and immune responses are the major component of secondary injury and play a central role in regulating the pathogenesis of acute and chronic SCI [3, 14]. Proteinases and, in particular, matrix metalloproteinases (MMPs), are likely mediators of early secondary vascular pathogenesis after SCI [136]. MMPs are soluble and cell-surface bound zinc-dependent endopeptidases that mediate cellular infiltration, extracellular matrix degradation and release of growth factors and cytokines from the matrix, cell migration, tissue damage, remodeling, and repair [20, 132]. They are synthesized as inactive precursors, which limits their potentially destructive properties, and become activated upon removal of the propeptide [184]. The catalytic activities of MMPs are highly regulated at multiple levels, including transcription, secretion, zymogen activation, and inhibition by tissue inhibitors of metalloproteinases (TIMPs) [188]. In particular, most MMPs are rapidly upregulated by transcription in response to the exposure of a cell to growth factors, cytokines, chemokines, extracellular matrix (ECM) components and other transcriptional regulators. Transcription is followed by translation, and an MMP is first produced in its pro-form that requires subsequent conversion to the active enzyme. Free radicals, serine proteases and other activated MMPs are among the factors that convert a pro-MMP to its active enzyme. MMPs activity is required for the inflammatory cell infiltration that occurs following SCI and most likely contributes to early barrier disruption. The early inflammatory response involves an initial wave of infiltrating neutrophils, followed by migration of monocytes and macrophages into injured segment. Each of these inflammatory cells expresses MMPs, including MMP-2 (gelatinase A), MMP-8 (neutrophil collagenase), MMP-9 (gelatinase B), MMP-11 (stromelysin-3), and MMP-12 (metalloelastase). MMP-2 and -9 degrade types IV and V collagen, and thus have the potential to modify constituents of basal laminae [101, 178]. Expression of MMP-2 and -9 has been demonstrated throughout the nervous system during development [207] and also in disease states, such as painful nerve injury, injured spinal cord, and cerebral ischemia [45]. In the nervous system, these enzymes are directly involved in axonal outgrowth during development [220]. On the other hand, the aberrant expression of MMPs is involved in various disease processes, e.g., cancer metastasis and CNS disorders including multiple sclerosis, stroke, Alzheimer’s disease, and trauma [220]. Recently, various studies using experimental SCI model have demonstrated an involvement of MMPs in the posttraumatic events. In particular, a significant upregulation of MMPs was demonstrated in a mouse SCI compression model [215]. Moreover, it has been also clearly demonstrated that the pharmacological blockade of MMPs, limited to the first several days after SCI, improves locomotor recovery [78]. Recently, it has been pointed out that reactive oxygen and nitrogen species regulate MMP activity *in vitro*, and disrupt the balance of MMP activation and inactivation [58]. Melatonin exerts protective effects reducing SCI-induced MMP-9 and MMP-2 activity and expression.

Much of the damage that occurs in the spinal cord following traumatic injury is due to the secondary effects of glutamate excitotoxicity, Ca2+ overload, and oxidative stress, three mechanisms that take part in a spiraling interactive cascade ending in neuronal dysfunction and death [4, 64]. The role of reactive oxygen-induced damage to spinal cord lipids (i.e., lipid peroxidation) and proteins has been strongly supported in various studies [217]. Antioxidative mechanisms of melatonin as well as its metabolites have been suggested to be singlet oxygen quenching and free radical scavenging during prevention of lipid peroxidation [5, 139]. Decreasing oxidative stress reduces the amount of secondary damage due to trauma. The regulation of MMPs identifies another mechanism whereby the melatonin minimizes oxidative damage and inflammation involved in SCI. In the CNS, MMPs participate in injury and disease states. In brain and spinal cord injury, MMPs, including MMP-2 and MMP-9, contribute to early secondary pathogenesis by disrupting the blood-brain/spinal cord barrier and promoting inflammation [169, 170]. Consistent with other studies, we show that MMP-2, like MMP-9, is upregulated during SCI [78, 136]. Various combinations of proinflammatory molecules may provide the signal for induction of MMPs, especially MMP-9 activity. The anti-inflammatory activity of melatonin involves a significant downregulation of MMP-9 activity and a modest depression MMP-2 activity. Therefore, the inhibition of the MMP-2, and MMP-9 by melatonin is most likely attributed to the suppressive effect on TNF-α production. 

The inhibition of mitogen-activated protein kinase family members, including extracellular signal-regulated kinases ½ (ERK1/2), c-jun N-terminal protein kinases, and p38 kinases, is believed to be beneficial in a number of experimental models of neurodegenerative diseases, diabetes type II, bipolar disorders, stroke, cancer, sepsis, and chronic inflammatory disease. The protective effect of melatonin might largely be accounted for by inhibition of p38 and ERK1/2 MAPK. Evidence indicates that ERK1/2 and especially p38 play an important role in NO-mediated degeneration of neurons in the spinal cord following SCI [218]. Previous studies also showed that the expression of activated ERK1/2 and p38 MAPK in microglia/macrophages may play a key role in production of CNS inflammatory cytokines and free radicals, such as NO [110]. Inhibition of phosphorylation of p38 MAPK and to a lesser extent of ERK1/2 reduces iNOS mRNA expression and rescues neurons from apoptosis after acute traumatic SCI [218]. We also here report that 24 hr after SCI, melatonin treatment significantly inhibit HMGB1 expression. In this study, our research group demonstrate that melatonin treatment significantly reduced the SCI-induced spinal cord tissue alterations as well as improved motor function. 

Like other forms of neurotrauma, TBI involves multifactorial etiologies including free radical generation, which culminates in oxidative and nitrosative damage, disrupted macrocirculation and microcirculation in the vicinity of the injury, lymphocytopenia, opportunistic infections, perturbed sleep-wake cycles, suppression of nonspecific resistance, and toxicity caused by therapeutic agents. Together, these factors precipitate into the development of heterogenous clinical symptoms in the secondary phase. Melatonin has been adjudged as a protective agent against damage following TBI in several *in vitro* and *in vivo* studies [118]. Enhanced vulnerability of brain to such oxidative or nitrosative stress that escalates the damage following TBI may be handled more deftly by melatonin compared to other antioxidants. Infection and inflammation complicates TBI in surviving patients. Melatonin also aids in inhibition of pro-inflammatory cytokines and activation of adhesion molecules. It would consequently reduce lymphocytopenia and infections by opportunistic organisms. Moreover, the chronobiotic capacity of melatonin may also reset the natural circadian rhythm of sleep and wakefulness, which may serve as a major advantage of melatonin’s use as a therapeutic molecule after traumatic injury. It may also reduce the toxicity and enhance the efficacy of drugs used in clinical management of TBI such as steroidal or nonsteroidal anti-inflammatory agents, anti-ulcer agents, anti-psychotics/antidepressants, anti-epileptics and also anti-anemic drugs. Similar benefits of melatonin may be seen in other traumatic injuries to CNS as well. Finally, melatonin, not being restricted by the blood-brain barrier, reportedly reduces the contusion volume and stabilizes cellular membranes preventing vasospasm and apoptosis of endothelial cells that occurs as a result of TBI [118, 156].

## EFFICACY OF MELATONIN ON BRAIN ISCHEMIA/REPERFUSION

The integrity of the CNS is highly vulnerable to a transitory interruption of its blood supply; this is specially true of the brain which is susceptible to focal or global ischemia. Unless ischemia is promptly reversed, reperfusion produces further cerebral damage. Ischemia/reperfusion injury is a subset of CNS damage that is unfortunately common but also occurs with equal frequency in nonneural tissues. In the CNS, interruption of the blood supply followed by reperfusion may not always be a result of traumatic assault. 

Melatonin effectively attenuated ischemic brain injury *via *the Bcl-2-related survival pathway by increasing the expression of Bcl-2 [110] and Bcl-xL [93] in the ischemic brain. 

Several recent reviews [30, 155, 212] summarize the reports that have documented the neuroprotective effects of melatonin against ischemia/reperfusion injury to the brain. Ischemia (focal or global) causes extensive destruction of neural tissue and the primary objective in the clinic is to promptly reverse it. Procedures such as acute thrombolysis or defibrinogenation, however, are effective only in selective patients, and are associated with a significant risk of complications due to bleeding. To date, a number of neuroprotectants, which were initially found effective in experimental studies for stroke therapy, failed in clinical trials. *In vivo* and *in vitro* evidence in favor of the use of melatonin to protect against focal and global cerebral ischemia/reperfusion injury has been reviewed [212]. The benefits of conducting further experimental studies to examine any synergistic protective action by combining melatonin with thrombolysis, defibrinogenation or other neuroprotectants are emphasized. The planning of phase II and phase III trials in an attempt to define the potential benefits of melatonin as an acute stroke treatment in humans is suggested [212]. When the results of all the reports are considered, it is clear that endogenously-produced and exogenously-administered melatonin reduces the degree of tissue damage and limits the behavioral deficits associated with experimental models of stroke [155]. The most recent updates on melatonin’s efficacy in models of stroke come from another review where the potentially widespread applications of melatonin have been showed [30]. These studies provide the preclinical tests for melatonin in acute stroke therapy, and its application at different treatment schedules. Advantageous characteristics of melatonin as a neuroprotective drug relates to its bioavailability, its potential for exerting wide-spread neuroprotective actions, further amplified and prolonged by its metabolites [146], its ability to reduce inflammation, its capability to protect cytoskeleton organization, and its anti-apoptotic actions. Moreover, melatonin does not interfere with the thrombolytic and neuroprotective actions of other drugs. An adequate safety profile of the drug has been underscored. 

## EFFECT OF MELATONIN ON MIDDLE CEREBRAL ARTERY OCCLUSION (MCAO) MOUSE MODEL

Melatonin has proved effective not only in the cell-free purified mitochondrial system but also inhibits cytochrome *c *release in an middle cerebral artery occlusion (MCAO) mouse model [92, 97] and in primary cortical neurons (PCN) [97]. Thus, melatonin is likely to interfere with both caspase-dependent (cytochrome *c*) and independent (AIF) mitochondrial cell death pathways. *In vitro *and *in vivo *experiments have shown that melatonin prevents the activation of downstream caspase-3 in oxygen/glucose deprivation (OGD)-mediated PCN cell death [97], cerebral ischemia-induced mouse injury, and the MCAO rat model [82, 93, 97]. Other experiments demonstrate significantly fewer TUNEL-positive cells [88, 98], reduced levels of cleaved PARP, and less DNA fragments [92] are found with administration of melatonin in the rat MCAO model. In addition, melatonin prevents brain damage, with reduced TUNEL-positive cells following transient cerebral artery occlusion (CerAO) [187] and transient MCAO model [35], as well as attenuating kainic acid-induced neuronal death, and reduces the number of TUNEL-labeled DNA breaks [154].

The neuroprotective role of melatonin is also mediated through the enhancement of the PI3-K/Akt survival pathway [93, 98] and JNK pathway [93], and restores reduced phosphorylated Akt in a model of mouse intraluminal MCAO [93]. Melatonin protected neuronal cells from damage by enhancing the activation of Akt and its downstream target Bad, without affecting the expression of 14-3-3, which acts as an antiapoptotic factor through interaction with Bad, thus mediating antiapoptosis signals in a rat MCAO model [88]. Furthermore, in the same model, melatonin inhibits apoptotic signals by preventing the injury-induced decrease of phosphorylation of Raf-1, MEK1/2, and ERK1/2 and the downstream targets, including Bad and 90-kDa ribosomal S6 kinase [98].

## CONCLUSIONS

Based on the data summarized herein, the multifunctional molecule melatonin may be a useful therapeutic agent for treatment of CNS injuries [154] (Table **1**). There is a wealth of well-documented experimental research to support its use. Melatonin is safe, nontoxic, and available in pure form for human use as a drug. The results of experimental studies provide fundamental information for the effective design and execution of clinical trials using melatonin as a neuroprotective treatment for traumatic CNS injuries.

## Figures and Tables

**Table 1:** Efficacy of Melatonin in Animal Models of Neurodegenerative Disease and CNS Traumatic Injury

Diseases/Models	Effect	References
Neurodegeneration	Inhibits cytochrome *c* release from mitochondria Reduces number of DNA breaks	[141, 142]
MCAO	Decreases cytochrome *c* release Attenuates cerebral ischemic injury Prevents caspase-3 activation Displays decreased DNA Decreases TUNEL-positive cells	[221-223] [225]
Parkinson disease (PD)	Prevents cytochrome c release Prevents ΔΨm depolarization in astrocytes Prevents ROS formation Blocks caspase-3 activation Prevents DNA fragmentation Inhibits cell death in SK-N-SH cells	[128-130]
Huntington disease (HD)	Neuroprotective	
Amyotrophic lateral sclerosis (ALS)	Reduces ROS	
Alzheimer disease (AD)	Reducts Par-4 upregulation Blocks A*β*25-35-induced apoptosis Anti-inflammatory effect on A*β* vaccination in mice Improves spatial memory performance Protects the wortmannin-induced tau hyperphosphorylation	[110-124]
Brain ischemia/reperfusion	increases the expression of Bcl-2 and Bcl-xL in the ischemic brain	[217-219]
Spinal cord injury (SCI)		[158, 161, 164, 167, 168, 207, 208]
Traumatic brain injury (TBI)		[42, 45]
